# Longitudinal analysis of safety and medication adherence of patients in the Fingolimod patient support program: a real-world observational study

**DOI:** 10.1038/s41598-021-83220-1

**Published:** 2021-02-18

**Authors:** Aline Bourdin, Marie Paule Schneider, Isabella Locatelli, Myriam Schluep, Olivier Bugnon, Jérôme Berger

**Affiliations:** 1 Community Pharmacy, Center for Primary Care and Public Health (Unisanté), University of Lausanne, Lausanne, Switzerland; 2 School of Pharmaceutical Sciences, University of Geneva, Geneva, Switzerland; 3 Institute of Pharmaceutical Sciences of Western Switzerland, University of Geneva, University of Lausanne, Geneva, Switzerland; 4 DFRI, Center for Primary Care and Public Health (Unisanté), University of Lausanne, Lausanne, Switzerland; 5 Division of Neurology, Department of Clinical Neurosciences, Centre Hospitalier Universitaire Vaudois, Lausanne, Switzerland

**Keywords:** Multiple sclerosis, Health services, Patient education

## Abstract

The Fingolimod Patient Support Program (F-PSP) is an interprofessional specialty pharmacy service designed to ensure responsible use of fingolimod by promoting patient safety and medication adherence. This study aims to evaluate the safety and medication adherence of patients who joined the F-PSP between 2013 and 2016. Sociodemographic and medical characteristics, patient safety data (patient-reported symptoms, discontinuations due to adverse events (AEs), repeated first-dose monitoring), and medication adherence (implementation, persistence, reasons for discontinuation, influence of covariates, barriers and facilitators) were described. Sixty-seven patients joined the F-PSP. Patients reported a high frequency of symptoms. Due to AEs, 7 patients discontinued fingolimod, 3 took therapeutic breaks, and 1 reduced the regimen temporarily. Three patients repeated the first-dose monitoring. Patients had a high medication adherence over the 18-month analysis period: implementation decreased from 98.8 to 93.7%, and fingolimod persistence was 83.2% at 18 months. The patients’ level of education, professional situation, and living with child(ren) influenced implementation. Patients reported more facilitators of medication adherence than barriers. The F-PSP seems valuable for supporting individual patients (ensuring responsible use of fingolimod and inviting patients for shared-decision making) and public health (indirectly gathering real-world evidence).

## Introduction

Fingolimod is an oral disease-modifying therapy (DMT) that was approved as a first-line drug for relapsing–remitting multiple sclerosis (MS) in the USA, Switzerland, and Australia. Pharmacovigilance measures are mandatory due to safety issues such as cardiovascular, ophthalmic, hematologic, hepatic, or pulmonary complications and risk of infections, cancer, and fetal toxicity^1^. Six hours of medical monitoring for bradycardia is required immediately after administration of the first dose, and various other medical tests are recommended prior to and after treatment initiation. Patients should be warned about the symptoms of potential serious adverse events (AEs), and women of childbearing potential should be informed about the teratogenic risk and use of effective contraception^2^. Moreover, chronic drug intake is challenging^3^. Optimal DMT adherence is associated with a decreased risk of relapses and medical costs^4,5^. Therefore, support of patients taking fingolimod is encouraged^2,4^.

In 2013, the Community Pharmacy of the University Center for Primary Care and Public Health, Lausanne (Switzerland), and the MS clinic of the Lausanne University Hospital (Centre Hospitalier Universitaire Vaudois—CHUV), launched and disseminated in a pharmacy network an interprofessional specialty pharmacy service: the Fingolimod Patient Support Program (F-PSP)^6^. The F-PSP aims to ensure responsible use of fingolimod and empowerment of patients by promoting patient safety and medication adherence through a person-centered and integrated care approach. It consists of regular pharmacist-led motivational interviews supported by a secure web platform (from SISPha SA), which includes a clinical decision aid system, patient health records, and a data collection system (recording, e.g., patient administrative data, adherence data, patient-reported outcomes). Patient safety is ensured through pharmacovigilance activities by informing patients regarding the responsible use of fingolimod (e.g., missed dose management, active contraception, co-treatment management), remind them about recommended medical tests, and track and monitor patient-reported symptoms (especially those who would signal potential serious adverse fingolimod reactions). Fingolimod adherence is monitored by an electronic monitor (EM) (MEMS SmartCap, Aardex Group). The cap of the EM bottle in which fingolimod is supplied records each opening and displays it on an LCD screen as a real-time reminder for patients. At the pharmacy, EM data are uploaded onto a secure website. Patient intake data are displayed on a graph, which is used by the pharmacist to give feedback to the patients during each interview. To mitigate the risk of bias linked to EM use, its use is validated prior to each interview by a pill count and by asking patients whether fingolimod has ever been taken without EM (e.g., pocket doses). During interviews, barriers to and facilitators of medication adherence identified by pharmacists are recorded on the SISPha-platform. At the end of each interview, the platform issues a structured report, which is available to the patient’s neurologist, MS nurse, general practitioner, and/or other pharmacists to ensure continuity of care.

The F-PSP is disseminated throughout Western Switzerland through a trained pharmacy network (22 pharmacies, including the Pharmacy of Unisanté). Participation in the F-PSP is proposed during the first-dose monitoring to each patient starting fingolimod at the MS clinic. Patients are free to join in any network pharmacy and can withdraw at any time.

This study aims to evaluate the safety and medication adherence of patients participating in the F-PSP during the first three years post-implementation.

## Methods

The cantonal commission for the ethics of human research (*Commission cantonale d'éthique de la recherche sur l'être humain du canton de Vaud*—CER-VD) approved this observational study (2016–00328) and informed consent to participate in the study was obtained from all participants. This study was performed in accordance with that Ethics Committee relevant guidelines and regulations. The included population consisted of MS patients starting fingolimod and joining the F-PSP between October 2013 and October 2016.

### Patient sociodemographic and medical characteristics

Sociodemographic data were collected using a 6-item questionnaire. Medical data were extracted retrospectively from the records of the MS clinic. The sociodemographic and medical characteristics of F-PSP patients at the time of fingolimod initiation were compared to those of patients who did not join the F-PSP. Two-tailed Student’s *t* tests were used to compare data with normal distributions, and the Wilcoxon–Mann–Whitney test was applied for non-normally distributed data. Fisher’s exact test and the chi-square test were used to test for differences in categorical variables. Statistical significance was set at *p* < 0.05. Analyses were performed with Stata version 15.0 (StataCorp, College Station, Texas).

### Patient safety

Patient safety was assessed over the 3-year F-PSP observation period (2013–2016). Patient-reported symptoms during repeated F-PSP interviews and the level of discomfort assessed by patients were extracted from the SISPha-platform. A symptom was defined as a subjective experience reported by the patient^7^. The most frequent symptoms were those reported by ≥ 10% patients, a cutoff commonly used in drug monographies.

The repeated first-dose monitoring was established based on the medical records and was recorded on the SISPha-platform.

### Medication adherence

Adherence was operationalized through the definitions of implementation and persistence as described by the ABC taxonomy. Implementation is defined as “the extent to which a patient’s actual dosing corresponds to the prescribed dosing regimen, from initiation until the last dose is taken”^8^. Implementation on day D is the probability of taking (at least) all prescribed doses on day D for a subject who is still persistent on that day^9^. Persistence is the time between initiation and discontinuation of the treatment^8^. Persistence on day D is the probability of not yet having discontinued on day D^9^. Discontinuation is either a unilateral patient decision or a physician–patient joint decision (e.g., AEs, ineffectiveness or pregnancy). Adherence on day D is the probability of taking at least all prescribed doses on day D (for a patient who entered the F-PSP)^9^. Implementation and persistence were modelled longitudinally using EM data.

#### Data validation

EM raw data were extracted from the SISPha-platform and validated in sets. A data set included all opening dates, which occurred between two consecutive F-PSP interviews. Each data set was validated by calculating the absolute difference (ABS) between the electronic drug monitoring-based proportion of prescribed doses taken (%EDM) and pill count-based proportion of prescribed doses taken (%PC) for each inter-visit period as follows: ABS (%EDM—%PC)^10^. Set data were considered as valid if the difference was < 25%. This cutoff was defined based on the experience of the group^11,12^; a higher difference would substantially decrease the validity of the adherence measure. If it was ≥ 25%, the %EDM was recalculated by including periods of pocket dosing reported by the patient. If the difference was still ≥ 25%, %EDM and %PC were recalculated by merging the following intervisit period. If that was not possible, the %EDM was compared with the previously validated %EDM and was considered valid when the difference was within a range of ± 10%. Data sets with a ≥ 25% difference were excluded from the analysis.

#### Data analysis

The longitudinal analysis was performed over the first 18 months of treatment, defined by the median observation time (from fingolimod initiation to the last EM opening) to ensure sufficient data for a reliable analysis. Each pocket dose was considered a missing value. Openings made from midnight to 2 h 59 were considered to belong to the previous day. Extra daily openings were ignored. Short breaks (≤ 1.5% of total days—arbitrary cut-off of individual total monitored days) in treatment prescribed by the neurologist (due to AEs) were considered missing values. In cases of long breaks, only the first data sets were analyzed and patients were considered to have stopped treatment according to a physician–patient joint decision on the date of the beginning of the break.

Implementation was computed at each day D as the proportion of patients taking fingolimod as prescribed among the persistent patients and not censored on day D. Patients were censored if they withdrew from the F-PSP before the end of the analysis period but continued taking fingolimod. A logistic generalized estimating equation model (exchangeable correlation) was used to estimate the implementation over time using polynoms of time as covariates. Persistence was represented by a Kaplan–Meier curve, which estimates the probability of being persistent on each day D, taking into account censoring durations. Adherence was calculated on each day D as the product between implementation and persistence, representing the probability of taking at least the prescribed dose on day D^9^.

### Covariate influence

The influence of covariates on implementation was assessed using univariate adjusted analyses. The covariates, measured at fingolimod initiation, were age (< 35/ ≥ 35 years), gender (men/women), level of education (low (primary and secondary levels)/high (tertiary level)), professional situation (active/inactive), living with child(ren) (yes/no), previous DMT (yes/no) and disease activity (≤ 1/ > 1 relapse).

### Reasons for discontinuation

Reasons for discontinuation were identified based on the information reconciled by the SISPha-platform and the medical records. Barriers to and facilitators of adherence were extracted from the SISPha-platform. Both outcomes were assessed over the 3-year F-PSP observation period (2013–2016).

## Results

**Figure 1:**
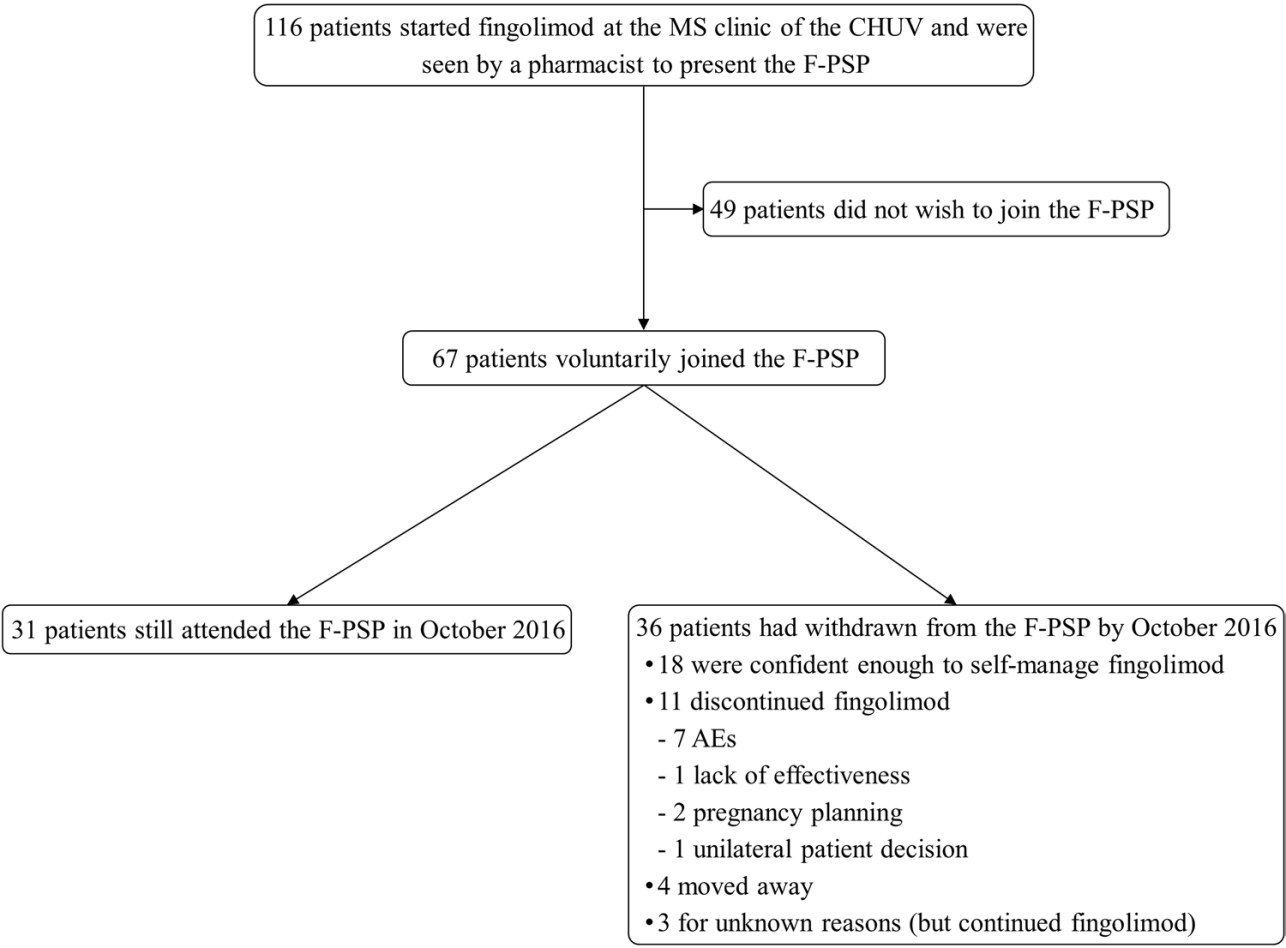
F-PSP status and number of patients over the 3-year observation period (Oct. 13 to Oct. 16); CHUV: Centre Hospitalier Universitaire Vaudois (Lausanne University Hospital); AE: adverse event; F-PSP: Fingolimod Patient Support Program; MS: multiple sclerosis.

From October 2013 to October 2016, 116 patients started fingolimod at the CHUV and were seen by a pharmacist for inclusion in the F-PSP; 58% (67/116) patients voluntarily joined the program in 11 network pharmacies (Fig. 1). Questions asked by patients during the F-PSP presentation are presented in the Additional file.

Over this 3-year period, pharmacists conducted 553 F-PSP interviews with the 67 patients, with an average (mean ± standard deviation) of 8.3 ± 4.2 interviews/patient. The median patient retention time in the F-PSP was 11 months (interquartile rage (IQR): 6–19)).

### Patient sociodemographic and medical characteristics

Table 1 presents the patients sociodemographic and medical characteristics. No differences in sociodemographic characteristics were observed between the F-PSP patients and those who did not join the F-PSP. However, differences were observed regarding the medical characteristics: F-PSP patients had a significantly higher annualized relapse rate before fingolimod initiation, a shorter disease duration, and no previous DMT compared with patients who did not join the F-PSP.

**Table 1:** Sociodemographics and medical characteristics of F-PSP patients and of patients who did not join the F-PSP at the time of fingolimod initiation. AAR: annualized relapse rate; DMT: drug modified therapy; EDSS: Expanded Disability Status Scale; IQR: interquartile range; n: number; SD: standard deviation. ^a^Nine patients could not be interviewed with the sociodemographic questionnaire and 1 refused it; ^b^For example, stay-at-home parents, students, unemployed, retired, disability insurance, on maternity leave; ^c^Eight patients did not give consent to access their medical records; ^d^Chi-square test; ^e^Student’s t-test; ^f^Fisher's exact test; ^g^Wilcoxon–Mann–Whitney test.

	All	F-PSP patients	Patients not joining F-PSP	*P*
Patients—n (%)	116 (100)	67 (58)	49 (42)	
Female—n (%)	76 (66)	42 (63)	34 (69)	0.453^d^
Male—n (%)	40 (34)	25 (37)	15 (31)	
Age, years—mean (SD)	38 (12)	37 (12)	39 (11)	0.292^e^
**Sociodemographic data**^**a**^	**106** (**91**)	**62** (**93**)	**44** (**90**)	
Level of education—n (%) *2 missing values*				
Primary level	8 (8)	5 (8)	3 (7)	0.867^f^
Secondary level	47 (45)	26 (43)	21 (49)	
Tertiary level	49 (47)	30 (49)	19 (44)	
Professional situation—n (%)				
Working	74 (70)	43 (69)	31 (70)	0.842^f^
Not working^b^	32 (30)	19 (31)	13 (30)	0.903^d^
Activity of working patients—n (%)				
Full-time (100%)	35 (47)	21 (49)	14 (45)	0.755^d^
Part-time (< 100%)	39 (53)	22 (51)	17 (55)	
Home situation—n (%)				
Alone	21 (20)	10 (16)	11 (25)	0.532^f^
With a partner	64 (60)	37 (60)	27 (61)	
With parent(s)	14 (13)	10 (16)	4 (9)	
With roommate(s)	7 (7)	5 (8)	2 (5)	
Living with child(ren)—n (%) *1 missing value*				
Yes	48 (46)	27 (44)	21 (48)	0.725^d^
No	57 (54)	34 (56)	23 (52)	
**Medical data**^**c**^	**108** (**93)**	**66** (**99**)	**42** (**86**)	
EDSS—median (IQR)	1.5 (1.5–2.0)	1.5 (1.5–2.0)	1.5 (1.5–2.4)	0.772^g^
ARR before fingolimod initiation—median (IQR)	0.6 (0.3–1.5)	0.8 (0.4–2.1)	0.5 (0.3–0.8)	0.018^g^
Months since diagnosis—median (IQR)	24 (4–69)	8 (3–42)	47 (19–116)	< 0.001^g^
Previous DMT—n (%)				
Yes	55 (51)	25 (38)	30 (71)	0.001^d^
No	53 (49)	41 (62)	12 (29)	

### Patient safety

The 67 patients reported 965 symptoms (97% of patients reported any symptoms), 56% were assessed as mild, 32% moderate, and 11% major discomfort (10% missing values). Table 2 presents the most frequently reported symptoms.

**Table 2:** Most frequent symptoms reported by ≥ 10% patients (n = 67) during the F-PSP interviews (n = 553) and number of patients who reported symptom(s) of a potential serious adverse fingolimod reaction from 2013 to 2016. n: number. ^a^At least two cumulative symptoms: fatigue; nausea; vomiting; fever; joint/muscular pain; shivering; herpes; shingles; ^b^At least one of the following symptoms: blurred, distorted, or veiled vision; shaded or black spot in the center of vision; hypersensitivity to light; change in color perception; ^c^At least two cumulative symptoms: fatigue; dizziness; palpitations; ^d^At least two cumulative symptoms: fatigue; nausea; vomiting; epigastric pain; loss of appetite; yellow skin or eyes; dark urine; ^e^Breathing disorders.

	Patients n (%)	Interviews n (%)
Any symptom reported	65 (97)	348 (63)
**Most frequent symptoms reported by ≥ 10% patients**		
Fatigue	48 (72)	205 (37)
Dizziness	32 (48)	71 (13)
Headaches	32 (48)	78 (14)
Joint/muscular pain	30 (45)	69 (12)
Blurred or distorted or veiled vision	28 (42)	62 (11)
Palpitations	22 (33)	41 (7)
Diarrhea	17 (25)	33 (6)
Nausea	16 (24)	34 (6)
Epigastric pain	16 (24)	29 (5)
Breathing disorders	16(24)	27 (5)
Coughing	16 (24)	23 (4)
Hypersensitivity to light	15(22)	28 (5)
Back pain	13 (19)	21 (4)
Influenza Viral Infections	12 (18)	15 (3)
Dark urine	10 (15)	16 (3)
Shivering	10 (15)	15 (3)
Loss of appetite	9 (13)	15(3)
All other infections	9 (13)	12 (2)
Vomiting	7 (10)	11 (2)
**Potential serious adverse fingolimod reactions**		
Infections^a^	39 (58)	–
Macular edema^b^	37 (55)	–
Cardiovascular disorder events^c^	35 (52)	–
Hepatic disorder events^d^	31(46)	–
Pulmonary disorder events^e^	16 (24)	–

Forty-four (66%) patients reported at least one symptom classified as one of the most common AEs (influenza viral infections, headaches, coughing, diarrhea or back pain) of fingolimod. Table 2 summarizes the number of patients who reported symptom(s) of a potential serious adverse fingolimod reaction.

Table 3 describes the consequences of AEs and the number of patients who had to repeat the first-dose monitoring plan after a treatment break.

**Table 3:** Adverse event consequences and repeated first-dose monitoring (n = 67 patients). AEs: adverse events; EOD: every other day; n: number.

	Patients n (%)	Timing (in months) and length (in days)
**AEs consequences**		
Fingolimod discontinuation due to AEs	7(10)	2nd, 4th, 8th [2 patients], 13th [2 patients], 20th
Fingolimod breaks	3 (4)	4th (8 days), 10th (104 days), 23rd (77 days)
Fingolimod regimen reduced to EOD	1 (1)	7th (31 days)
**Repeated 1st dose monitoring**		
Following fingolimod break	2 (3)	13th, 26th
Following haphazard drug intake	1 (1)	6th

### Medication adherence

EM data were validated for 50/67 (75%) patients. Validation could not been perform in 16 patients because pill count values were missing throughout their follow-up. Additionally, half of the EM data was invalid for another patient despite the reconciliation process. According to reports from the F-PSP interviews, the latter did not use the EM systematically. Therefore, this patient was excluded from the adherence analysis. In addition, the data set of one intervisit period (127 days) for one patient was not valid, and these data were considered missing values.

A sensitivity analysis of patients with validated EM data vs those with unvalidated data showed no difference between the two groups, which allowed for data pooling (50 plus 16 patients) for the analyses.

**Figure 2:**
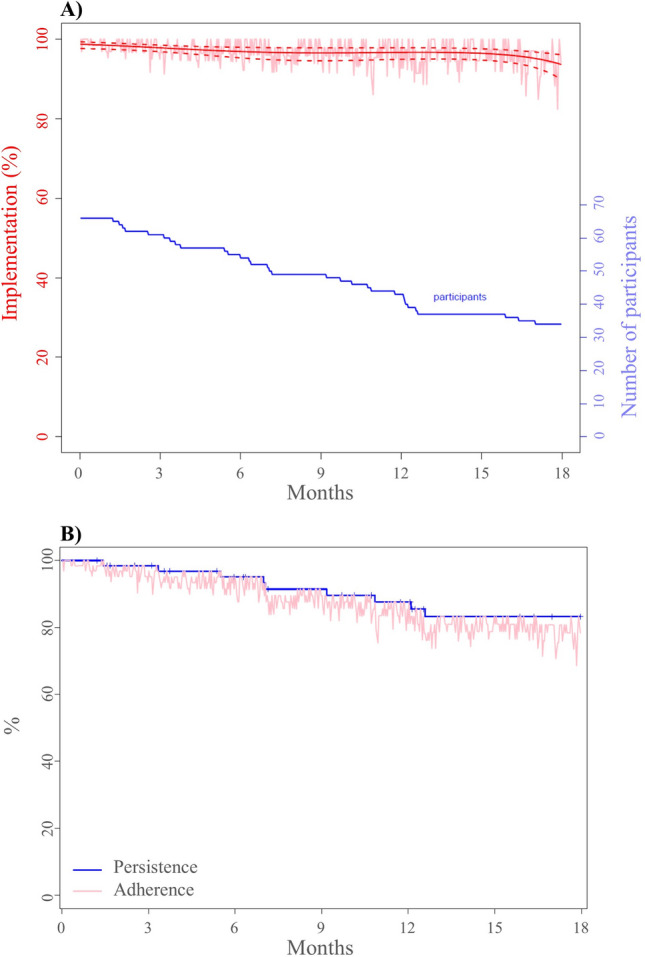
(**A**) Implementation among patients taking fingolimod and number of participants over 18 months, and (**B**) Persistence and adherence estimations among patients taking fingolimod over 18 months.

Implementation (95%CI) decreased over time, from 98.8% (97.7%–99.3%) at initiation to 93.7% (89.9–96.1%) at 18 months (Fig. 2A). Figure 2B shows that 83.2% (73.7%—94.0%) of the patients were still persistent after 18 months of treatment.

Over the 3-year period of F-PSP observation, 11 (16%) patients discontinued fingolimod: 10 (91%) based on physician–patient joint decisions, and one (9%) according to a unilateral patient decision. Table 4 presents the reasons for fingolimod discontinuation and switching to other DMTs.

**Table 4:** Reasons for fingolimod discontinuation and switching to other DMTs (n = 67 patients). DMT: drug modified therapy; IFN: interferon; n: number; SC: subcutaneous; ^a^Two patients were lost to follow-up after discontinuation, so information on the switch is missing.

	Patients n (%)	Timing (in months)
**Reasons for discontinuation**	**11 (16)**	
Adverse events	7 (64)	2nd, 4th, 8th [2 patients], 13th [2 patients], 20th
Pregnancy planning	2 (18)	11th, 20th
Lack of effectiveness	1 (9)	25th
Unilateral patient decision	1 (9)	6th
**Switches**^**a**^	**7 (64)**	
Dimethyl fumarate	3 (27)	7th, 16th, 25th
Natalizumab	3 (27)	12th, 20th, 25th
INF-Bêta1a SC	1 (9)	9th

**Figure 3:**
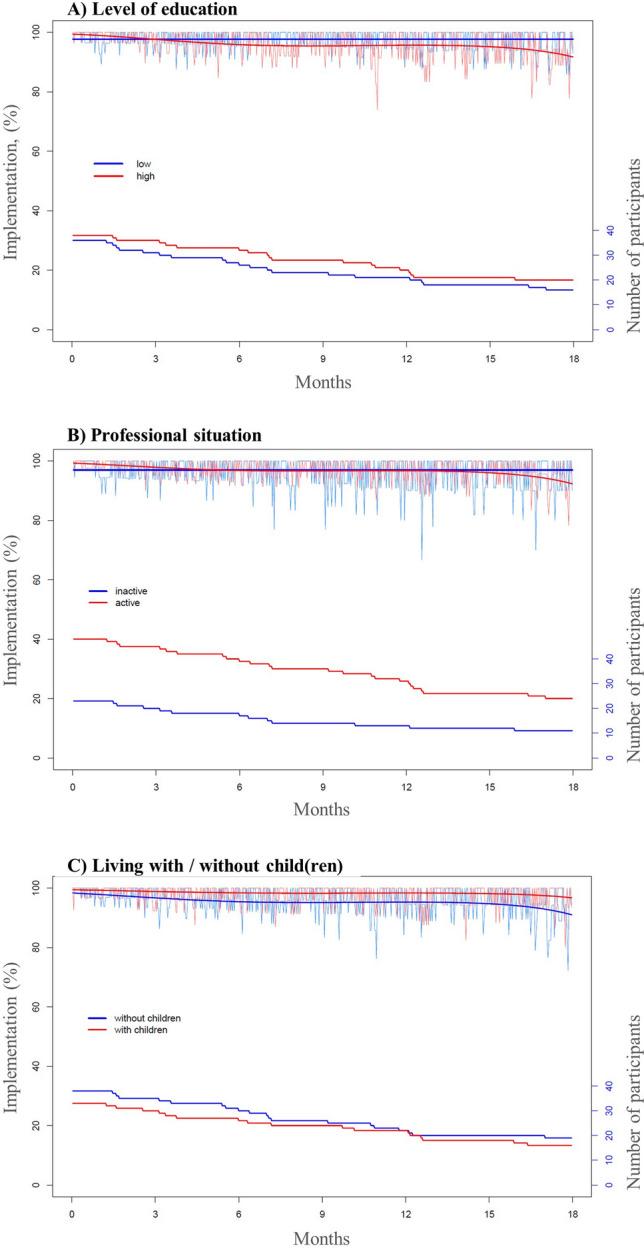
Influence of the level of education (**A**), professional situation (**B**), and living with child(ren) (**C**) on implementation by patients taking fingolimod.

Level of education, professional situation, and living with child(ren) influenced implementation, but the other tested variables did not (Fig. 3). Fingolimod implementation of patients with a high level of education or who were professionally active decreased over time, while time had no influence on the implementation of less educated or professionally inactive patients (straight line in Fig. 3A,B). Patients living with child(ren) had higher implementation than patients living without child(ren) over the entire time period.

**Figure 4:**
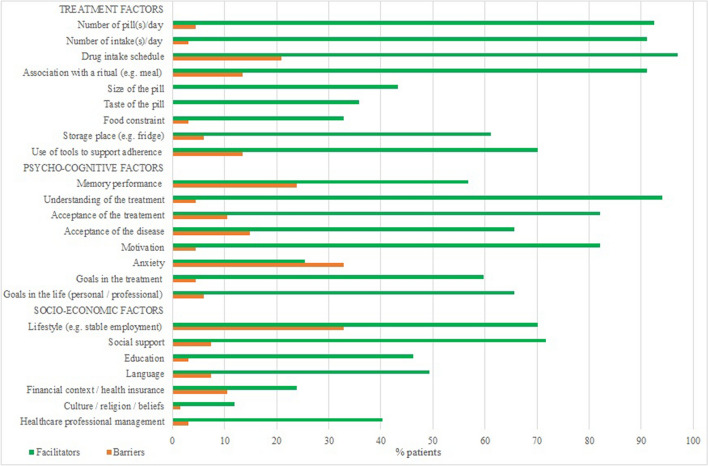
Facilitators of and barriers to fingolimod adherence by F-PSP patients.

All F-PSP patients had at least one facilitator of adherence during their follow-up, and 76% of patients had at least one barrier. The main facilitators of each single following category were as follows: drug intake schedule (97% patients) as a treatment-related factor, good understanding of the treatment (94%) in a psycho-cognitive factor, and social support (77%) as a socio-economic factor. The main barriers of each category were the drug intake schedule (21%), anxiety (33%), and an unstructured lifestyle (33%), respectively (Fig. 4).

### Discussion

This study assessed the safety and medication adherence of 67 patients taking fingolimod who entered the F-PSP. The sociodemographic characteristics of those patients were similar to those who did not join the program. However, DMT-naïve patients with high annualized relapse rates and short disease durations were more likely to join the program than their counterparts. Thus, the patients most likely to accept this program were those in a more acute situation of their disease and/or at a time just after diagnosis of their disease and/or receiving their first treatment.

Almost all patients reported at least one symptom during their F-PSP follow-up. The proportion of patients who reported symptoms were higher than those documented in clinical trials^13,14^ and other real-world clinical practice studies^15–18^. Compared with the rates reported in clinical trials, blurred vision occurred greater than ten times more frequently, fatigue and dizziness more than six times, and diarrhea and headaches more than two times^1,13,14^. More than half of the patients reported symptoms of potential serious adverse fingolimod reactions: infections, macular edema, and cardiovascular disorder events. However, symptoms reported are probably not all due to these serious adverse reactions; medical exams should be performed to confirm such diagnosis. Patients reported symptoms more frequently than in other real-world clinical practice studies, most likely because the F-PSP interviews focus on patients’ experiences, concerns, and beliefs. They were not objectified by a physician or a medical test. In addition, our data were from a pharmacy-based patient support program, with more frequent visits than those to the physician; thus, the patients are more likely to remember all their symptoms.

AEs resulted in fingolimod discontinuation in 10.4% of the study sample over a wide range of months (min–max: 2nd-20th with the majority within the first 13 months). This latter finding is inferior to those reported in Hersh et al*.* (13.1% over 12 months of follow-up)^16^ and Arichon et al. (16.4% over 12 months of follow-up)^18^, but superior to those in clinical trials^13,14^ and other real-world clinical practice studies^19–22^. The ability to make comparisons among these studies is limited because the majority had a shorter follow-up period than our study. Some studies focused on experienced patients (with at least 12 months of treatment), hence excluding those who could have discontinued fingolimod within the first year of treatment. AEs have drove 4.5% of fingolimod breaks, of which 2/3 led to repeated first-dose monitoring. Similarly to our results, in a large real-world clinical practice study assessing 2399 patients attending an educational support program (reinforcing adherence to medical tests but not to fingolimod), 3.9% of patients repeated the first-dose monitoring following a therapeutic break^23^.

Implementation was high but decreased over time (from 98.8 to 93.7%). This high implementation can be ascribed to the tailored support provided by the program. Indeed, tools for self-monitoring, EM-feedback, and cognitive-educational interventions focused on supplying patients with knowledge, counseling, and accountability (all of which are F-PSP core elements) to positively influence medication adherence^24–26^. Another real-world study investigated fingolimod implementation (generalized estimating equation analysis using pill count data reconciled with refill data and periods of pocket dosing) in 98 patients over 2 years^27^. The mean estimated implementation rate was 98.6% (95% CI 98.5–98.7), and it was stable over time. This result was similar at 18 months, which is slightly higher than ours. This minor difference may be explained by the different measurement methods because nonelectronic measures may overestimate medication adherence^28^.

Our 18-month estimated persistence of 83.2% F-PSP patients is consistent with the results of a meta-analysis (25 real-world studies), which estimated the persistence rate of fingolimod as 82% (95% CI 79–85) at 1 year^29^. This finding is also in line with clinical trials (81.2% after 2 years)^13^ and with a phase IV trial (81.3% after 48 weeks)^30^.

Most fingolimod discontinuations (91%) were based on a physician–patient joint decision over the 3-year F-PSP observation period. Zimmer et al*.* reported a similar finding (89.5% in 98 patients followed over 2 years)^27^. Physician–patient joint decisions regarding discontinuation among patients attending the F-PSP were mainly associated with AEs, followed by pregnancy planning and ineffectiveness. Ineffectiveness and AEs were also the main reasons for discontinuing fingolimod cited by a systematic review based on real-world studies^31^.

Level of education, professional situation, and living with child(ren) influenced fingolimod implementation. Although the literature describes positive associations between adherence, education, and health literacy, our results add nuances to these findings^32^. Indeed, the implementation in this subgroup decreased over time, which was opposite to the trend observed in patients with lower levels of education. A hypothesis is that patients with a high level of education are likely to take progressive liberties in drug intake, realizing that occasional missed doses over time are inconsequential. Further investigations must investigate this finding.

While the structured lifestyle provided by patients’ professional activity seems to positively influence medication adherence in the early stages, the positive effect vanishes with time. Notably, this finding should be cautiously interpreted due to the unbalanced proportions of patients in the two subgroups.

Implementation was higher in patients living with child(ren). The presence of child(ren) may provide a more organized routine and structured lifestyle, facilitating drug intake. Moreover, children are probably a substantial source of motivation to take fingolimod with the hope to stabilize the disease over the long-term.

F-PSP patients reported a larger number of facilitators of fingolimod adherence than barriers, which corroborates the group’s high level of medication adherence. Anxiety was the factor most frequently identified by pharmacists as a barrier to taking fingolimod, which is known to be a burden for MS patients^33^.

One of the most substantial strengths of this study is the joint provision of relevant information regarding the safety and medication adherence of patients taking fingolimod in a real-world setting. Postmarketing, patient-reported data are needed to validate clinical trial findings in larger and unselected populations. Another strength is the longitudinal adherence assessment by EM measurement, which is considered the gold standard for measuring implementation accurately^34^. The 18-month median observation period of longitudinal data is longer than in most studies. In addition, the reconciliation of EM data with pill count data and periods of pocket dosing validated by the patients increases the reliability of the results^34^. The use of a persistence model including censures is another strength. The main limitation is the observational and retrospective nature of the study. In the absence of a control group, no inference can be drawn from it; the effectiveness or impact of the F-PSP could not be demonstrated. This should be the subject of another study, where participants would be observed over a same period of time and where adverse events would be collected similarly from patients in and out of the program. Generalization to the MS population is limited because we cannot infer that patients attending a support program are similar to the entire MS population. Patients attending the program probably represent the population who is more in need of support for initiating a treatment for the very first time. However, healthcare professionals should not miss experienced patients who have difficulties adhering to their treatment. Special attention should be provided to them to include them in such a program. In addition, AE imputation of patient-reported symptoms was not assessed; these were patients’ perceptions, which may not be associated with fingolimod. Finally, the validation of one-third of the EM data could not be performed because EM use was not sufficiently documented in some network pharmacies.

## Conclusion

The pharmacy-based F-PSP, with a pharmacovigilance activity in tracking patient-reported symptoms and monitoring of medication adherence, has an interest in supporting individual patients, ensuring responsible use of drugs, and an interest in public health, gathering valuable real-world evidence. The high frequency of patient-reported symptoms, including a few potential serious adverse fingolimod reactions, and the various AE consequences, highlight the importance of regular and repeated patient support over time. Symptoms, as subjective experiences perceived by patients, show the need to reassure patients and answer their questions. Moreover, F-PSP patients had high fingolimod implementation and persistence. The strengthening of medication adherence through this type of interprofessional program (pharmacist, neurologist, MS nurse, general practitioner) might eventually improve the therapeutic effect and reduce frequency of medical consultations. Pharmacovigilance and medication adherence monitoring have the potential to positively influence the overall cost of care per patient. All these findings favor expanding the dissemination of the F-PSP to other pharmacist-physician collaborations and to more patients starting fingolimod. Finally, the F-PSP should serve as a model for other specialty drugs, diseases, or healthcare contexts. To achieve this goal, further research in implementation science with more patients and interprofessional collaborations is needed, in conjunction with sound cost-effectiveness analyses.

## Supplementary Information

Supplementary Information.
