# Supplementary material for: Longitudinal analysis of safety and medication adherence of patients in the Fingolimod patient support program: a real-world observational study
Source: Sci Rep. 2021 Feb 18;11:4107. doi: 10.1038/s41598-021-83220-1 (PMC7892872; doi:10.1038/s41598-021-83220-1)
Supplement: Supplementary file 1 — Supplementary Information. [file 41598_2021_83220_MOESM1_ESM.docx]

Additional file: Types of questions (n=241) asked by 116 patients during the F-PSP presentation (at the time of fingolimod initiation).


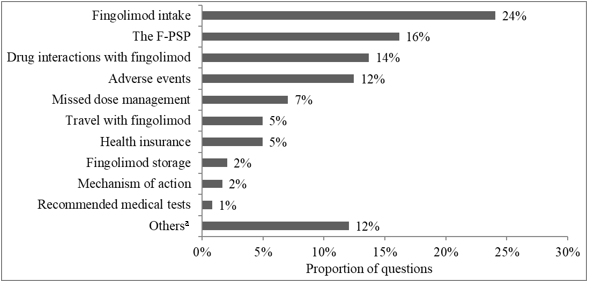


^a^

F-PSP: Fingolimod Patient Support Program

*^a^For example, questions related to contraception, cost, obtaining drug in other countries, and alternative medicine, among others.*
